# Synthesis and Characterization of Sulfonated Graphene Oxide Reinforced Sulfonated Poly (Ether Ether Ketone) (SPEEK) Composites for Proton Exchange Membrane Materials

**DOI:** 10.3390/ma11040516

**Published:** 2018-03-28

**Authors:** Ning Cao, Chaofan Zhou, Yong Wang, Hong Ju, Dongyang Tan, Jin Li

**Affiliations:** College of Mechanical and Electronic Engineering, China University of Petroleum (East China), Qingdao 266580, China; z16040387@s.upc.edu.cn (C.Z.); wangyong@upc.edu.cn (Y.W.); juhong@upc.edu.cn (H.J.); z16040373@s.upc.edu.cn (D.T.); s15040580@s.upc.edu.cn (J.L.)

**Keywords:** sulfonated poly (ether ether ketone), sulfonated graphene, proton conductivity, thermal stability, proton exchange membrane

## Abstract

As a clean energy utilization device, full cell is gaining more and more attention. Proton exchange membrane (PEM) is a key component of the full cell. The commercial-sulfonated, tetrafluoroethylene-based fluoropolymer-copolymer (Nafion) membrane exhibits excellent proton conductivity under a fully humidified environment. However, it also has some disadvantages in practice, such as high fuel permeability, a complex synthesis process, and high cost. To overcome these disadvantages, a low-cost and novel membrane was developed. The sulfonated poly (ether ether ketone) (SPEEK) was selected as the base material of the proton exchange membrane. Sulfonated graphene (SG) was cross-linked with SPEEK through the elimination reaction of hydrogen bonds. It was found that the sulfonic acid groups and hydrophilic oxygen groups increased obviously in the resultant membrane. Compared with the pure SPEEK membrane, the SG-reinforced membrane exhibited better proton conductivity and methanol permeability prevention. The results indicate that the SG/SPEEK could be applied as a new proton exchange membrane in fuel cells.

## 1. Introduction

Currently, the rapid growth of energy consumption and related environmental problems places a high demand on the development of the clean energy and clean power device [1]. Proton exchange membrane fuel cell (PEMFC), as a new energy conversion device, is gaining more and more attention because of its advantages of high efficiency, low cost, and environmental-friendliness [2]. Against this background, the research and development of proton exchange membranes (PEMs), as the “heart” of PEMFC, are of great significance [3], and it is a crucial factor deciding whether the PEMFC can be used as an economical and feasible energy. PEMs have dual functions of conducting protons and isolating positive and negative electrodes in PEMFC. Besides, PEMs applied to PEMFC must have outstanding hydration ability, as well as good mechanical properties, so as to avoid local water depletion and proton conduction reduction. In recent years, many studies have been devoted to the development of fluorinated and non-fluorinated acid ionomer PEMs, such as polystyrene-based membranes, sulfonated polyimide-based membranes, polyphosphazene-based membranes, polybenzimidazole-based membranes, and sulfonated aromatic polymer membranes [4]. Unfortunately, these membranes have not been used in practical applications due to the lack of hydrophilic groups or the poor hydrolysis stability of the polymer [5]. One of the commercial perfluorinated polymer PEMs named Nafion^TM^ was discovered in the late 1960s by Walther Grot [6]. The membrane was synthesized through polytetrafluoroethylene (PTFE) sulfonation with excellent chemical stability and electrochemical properties [7]. Moreover, it can maintain higher proton conductivity in a relatively dry environment, since there is both a hydrophobic region of PTFE skeleton and a hydrophilic zone of the sulfonic acid group [8]. However, it has many shortcomings in actual operation, such as high permeability, high cost, synthesis difficulty, and pollution [9].

The PEMs, which can meet the practical application, must have proper chemical stability, proton conductivity, and thermal stability [10]. However, it is difficult to realize these requirements at the same time. A lot of research has been devoted to the modification of PEMs. The organic/inorganic hybrid method can integrate the advantages of several materials to obtain new materials [11]. According to the interaction of organic-inorganic phase, the new materials can be divided into two categories [12]: (1) materials characterized by combination with secondary bonds, where the interfacial interactions are generally derived from relatively weak bonding, such as hydrogen bonds and van der Waals force; (2) material characterized in combination with chemical bonds, such as covalent bonds, ionic bonds, and coordination bonds. In the design of such a hybrid membrane, the polymer was used as a matrix, and the inorganic particles were filled as reinforcement fillers [13,14]. We intend to utilize the electrostatic interactions and the H-bonding between polymers and inorganic fillers to construct the microstructure between the interfaces of materials. The polymer matrix has good corrosion resistance and excellent chemical compatibility. The fillers increased the water retention and thermal stability of the hybrid membrane [15]. The obtained composite membrane has better chemical and thermal stability than the polymer membrane. Above all, the proton conductivity can be effectively improved under the condition of relatively low fuel permeability.

As a substitute of Nafion^TM^ for PEMs application, sulfonated poly (ether ether ketone) (SPEEK) has gained considerable attention due to its low cost, superior chemical stability, and excellent alcohol resistance [16]. In the process of SPEEK, sulfonic acid group (−SO_3_H) is introduced into PEEK molecules, and the resultant products exhibit a low fuel permeability [17]. It is found that the PEMs performance of SPEEK was closely related to the degree of sulfonation (DS) [18]. At higher DS, the PEMs exhibited higher proton conductivity. However, the liquid fuel permeation was higher, and the resultant structure stability of the PEMs decreased at the same time. To overcome this defect, inorganic fillers were selected to limit the liquid fuel permeation and maintain the structural stability of the SPEEK PEMs without decreasing proton conductivity.

As a derivative of graphene, the typical two dimensional nano-sheet structure gives graphene oxide (GO) good mechanical properties and large surface area. There are a lot of oxygen-containing groups on the GO nanosheets, such as carboxyl, hydroxyl, epoxy, etc. [19,20,21]. These groups not only make GO hydrophobic but also can be modified to form various kinds of novel graphene derivatives. Among these derivatives, sulfonated graphene (SG) was recognized as an excellent inorganic filler of composite PEMs [22]. GO lacks proton conducting groups and the introduction of the sulfonic acid groups can increase the proton-conducting channels in the membrane matrix and enhance the water retention of the composite membranes [23]. In addition, the surface sulfonic acid group in SPEEK and the oxygen-containing functional group in SG nanosheet can form a strong interfacial interaction with hydrogen bonds, which can reduce the permeability of methanol, weaken the expansion of the membrane, and improve the thermal stability of the composite PEMs.

In this work, we used the organic/inorganic hybrid method to dope SG into SPEEK substrates and develop new SG-reinforced SPEEK composite PEMs. It exhibited good interface compatibility and dimensional stability. In addition, the hydrophility of the sulfonic acid groups provided a large number of proton-conducting channels for the composite membrane, which had significantly better proton conductivity than the SPEEK matrix while retaining the alcohol resistance and swelling resistance of the SPEEK membrane. The results showed that SG/SPEEK composite membranes exhibited excellent chemical stability, proton conductivity, and thermal stability. It was believed to have great application potential for PEMs.

## 2. Experimental

### 2.1. Materials

The PEEK was purchased from Changchun Jilin University Super Engineering Plastics Research Co., Ltd. (Changchun, China). Graphite powder (<20 mL) was purchased from Baichuan Graphite Co., Ltd. (Qingdao, China). The 1,3-propanesultone (C_6_H_6_O_3_S), sulfuric acid (H_2_SO_4_), sodium nitrate (NaNO_3_), hydrogen peroxide (H_2_O_2_), hydrochloric acid (HCl), potassium permanganate (KMnO_4_), dimethyl sulfoxide (DMSO), *N*,*N*-Dimethylformamide (DMF), *N*-Methyl pyrrolidone (NMP), sodium hydroxide (NaOH), potassium biphthalate, ethylene glycol, acetonitrile, acetone, methanol, ethanol, and chloroform were all analytical grade and used as-received without further purification. The phenolphthalein reagent (0.5%), methyl orange phenolphthalein reagent (0.5%), and methylene blue phenolphthalein reagent (0.5%) were self-made in laboratory as indicators. Milli-Q water (18 MX) was used in all experiments.

### 2.2. Preparation of SG and SPEEK

Graphene oxide was synthesized from graphite power by the modified Hummers method [24]. The SG was prepared by creating a mixture of GO (3.5 mg/mL) and 1,3-propanesultone (8.3 M) in DMSO solvent. The mixture was exfoliated using a horn-type sonotrode (Branson, Danbury, CT, USA, Ultrasonic-Homogenizer Sonifier II W-450 with a 4.8 mm microtip) for 30 min in an ice bath and subsequently heated at 120 °C for 48 h. The resulting SG matrix was separated by centrifugation at 11,500 rpm for 15 min and washed with water and ethanol (3 times each) before being dried in vacuum oven at 60 °C for 12 h.

The 15 g dried PEEK was firstly added in the water (250 mL) in a three-necked flask equipped with a mechanical stirrer at 50 °C. After several drops of H_2_SO_4_ were added to completely dissolve the PEEK, the mixture was stirred at 50 °C for 12 h. The resultant yellow solution was slowly diluted with 10 times the volume of water under vigorous stirring, and white flocculent precipitate was obtained. The precipitate was filtered and washed 4 times with deionized water until the pH value reached 7. Finally, the SPEEK was dried under vacuum at 80 °C for 24 h [25].

### 2.3. Preparation of Composite PEMs

The SG/SPEEK solutions were prepared by blending SPEEK and SG at weight ratio of 99:0, 99:1, 98:2, and 95:5 in DMF solvent, respectively. The solutions were laid up in the fume cupboard overnight to get defoamed. Meanwhile, the mixture of GO and SPEEK was prepared at weight ratio of 99:1 in DMF in the above mentioned way to be used as the control group. Then, the solution was poured onto the bottom of glass petri dish evenly and dried at 100 °C for 24 h in vacuum to form a membrane. The membrane was peeled off and soaked in 1 M HCl for 24 h to be fully protonated. Finally, the membranes were washed and stored in deionized water for further use [26]. All the membranes were numbered in Table 1.

### 2.4. Characterization

#### 2.4.1. Chemical Structure and Micro-Morphologies Characterization

Fourier transform infrared (FTIR) spectra were performed on a Thermo Scientific FTIR instrument (Nicolet NEXUS^TM^, Ramsey, MN, USA) in the range of 4000–400 cm^−1^. The X-ray photoelectron spectroscopy (XPS) measurements were recorded on a PHI Quantum 2000 Scanning ESCA Microprobe (Eden Prairie, MN, USA) equipped with a monochromatic Al Kα X-ray radiation source and a hemispherical electron analyzer. The morphology of composites proton exchange membranes was investigated by a field emission scanning electron microscope (FESEM, Hitachi S-4800, Hitachi, Japan). Transmission electron microscopy images were recorded by means of JEOL JEM-F200 instrument (Tokyo, Japan). The thermal stability of the composite membranes was characterized by a thermal gravimetric analyzer (TG/DTA6300, SII, Chiba, Japan) over the 30–900 °C range at a heating rate of 10 °C min^−1^ in nitrogen atmosphere. Differential scanning calorimeter (TA Instruments Thermal Analysis System Q20, New Castle, DE, USA) measurement was employed to study the thermal transition behavior of PEMs. The samples were preheated in air from 30 °C to 150 °C at 10 °C min^−1^ to remove moisture, cooled to 90 °C, then reheated from that temperature to 300 °C at 10 °C min^−1^ in air.

#### 2.4.2. Water Uptake, IEC, DS, and Dissolvability

The membranes were dried at 70 °C in vacuum condition for 22 h, and their weights were measured. Subsequently, the dried membranes were immersed in water at room temperature for 24 h. Then, filter papers were employed to absorb the excess moisture on the wet membrane surfaces, and the wetted membranes were weighed. The water uptake was expressed as the ratio of swollen weight to dry weight using Formula (1) [27]:

(1) $$\mathrm{Wateruptake}(\%)=\frac{W_{\mathrm{wet}}-W_{\mathrm{dry}}}{W_{\mathrm{dry}}}$$

in which W_wet_ (g) and W_dry_ (g) are, respectively, the weight of the fully hydrated and the anhydrous membranes.

The ion exchange capacity (IEC) of S-0 sample was determined by the classical back titration method. A certain weight of S-0 sample was measured and dissolved in DMF. Then, the free ion was titrated by a 0.02 M NaOH solution with phenolphthalein as the indicator. The IEC value was calculated with the Formula (2):

(2) $$\mathrm{IEC}=\frac{C_{\mathrm{NaOH}}\times V_{\mathrm{NaOH}}}{W_{\mathrm{SPEEK}}}$$

in which C_NaOH_ is the concentration of NaOH (M), V_NaOH_ is the volume of NaOH used in the titration (mL), and W_SPEEK_ is the weight of S-0 samples (g).

The sulfonation degree (DS) of the sample can be calculated according to the Formula (3) [28].

(3) $$\mathrm{DS}=\frac{288\times \mathrm{IEC}}{1000-80\times \mathrm{IEC}}$$

in which 288 is the molecular weight of the repeating unit of the raw polymer, and 80 is the molecular weight of the sulfonate group.

The dissolvability of the PEMs was observed with Chloroform, DMF, ethanol, DMSO, NMP, and acetone being selected as solvent. 0.01 g of each dried PEMs was blended with 1 mL of the solvent in test tube and water-bath heated at 35 °C for 2 h. After standing for 1 more hour, the dissolvability of the PEMs and the precipitation were observed.

#### 2.4.3. Proton Conductivity and Methanol Permeability

Two-electrode method was used to measure the proton conductivity of the membranes in the transverse direction [29]. The platinum electrode was used as a counter electrode, and the mixture of 1 M methanol and 0.5 M H_2_SO_4_ was used as the test electrolyte. A ModuLab XM impedance/gain-phase analyzer over the frequency range of 10 Hz to 1 MHz was used for the measurements. The conductivity was calculated by Formula (4).

(4) $$\delta =\frac{L}{{(R}_{1}-R_{2})\times S}$$

in which L is the thickness of the conducting membrane, R_1_ is the resistance created by the membrane, R_2_ is the resistance in the absence of membrane, and S is the surface area of the membrane.

The methanol permeability of all samples at room temperature was measured by cyclic voltammetry with 0.5 M H_2_SO_4_ as electrolyte via a previously reported method [30,31]. Electrochemical tests were carried out through two self-made diffusion cells. One of the cells was filled with 5 M methanol solution (A, 16 mL), and the other one was filled with 0.5 M H_2_SO_4_ solution (B, 200 mL). The membranes were sandwiched between the two cells. The test conditions were as follows: scanning voltage −0.1~1.2 V, scanning speed 30 mV/s, number of cycles 3. Methanol permeability was determined from the slope of the plot of methanol concentration in the receptor compartment versus time, using the following Formula (5). The methanol permeability of the membrane at different times was obtained.

(5) $$C_{B}(t)=\frac{A}{V_{B}}\frac{D_{K}}{L}C_{A}(t-t_{0})$$

in which D_K_ is the methanol permeability coefficient (cm^2^/s), C_A_ is the concentration of methanol in the compartment V_A_ (mol/cm^3^), C_B_ is the concentration of methanol in the compartment V_B_ (mol/cm^3^), A is the membrane area (cm^2^), and L is the membrane thickness (cm).

## 3. Results and Discussion

### 3.1. Structure Characterization

**Figure 1:**
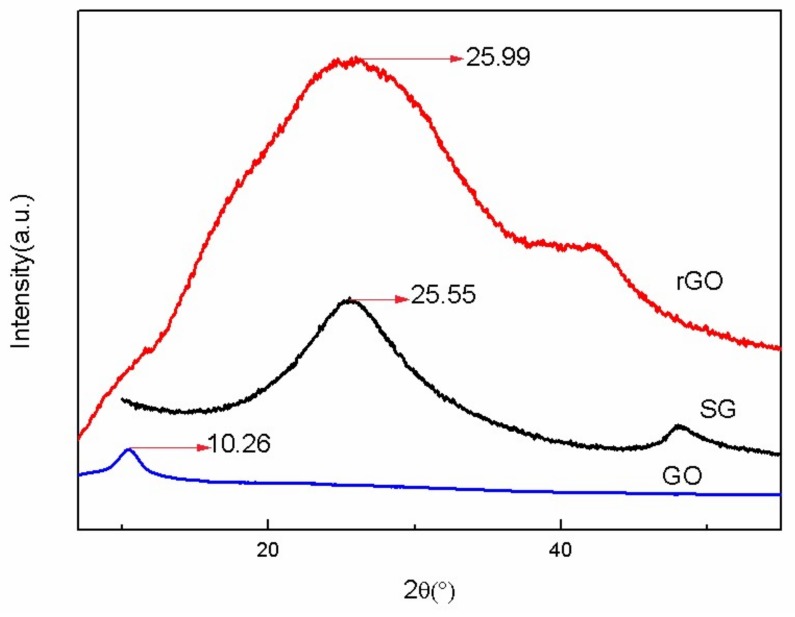
XRD spectra of GO, rGO, and SG.

The intercalation of SO_3_H into the rGO nanosheets and the resulting increase in the interlayer distance of rGO was confirmed by XRD analysis. Figure 1 compares the XRD patterns of the GO, rGO, and SG samples. In the case of GO, a sharp peak, which corresponds to the (001) plane, appears at 10.26°. The crystal planes and the average size of the ordered stacks of GO sheets can be calculated by using the well-known Debye-Scherrer formula as follows:

(6) $$L_{001}=K\lambda /\beta_{001}\mathrm{Cos}\theta$$

in which K, θ, λ, and β_001_, respectively, represent the shape factor, diffraction angle, the wavelength of the X-rays, and the full width at half maximum of the (001) peak of GO. The calculated results indicate that the thickness of the stack is 2.0 ± 0.2 nm, which corresponds to about 2 to 3 single atomic layers of GO sheets.

In the case of SG (Figure 1), two diffraction peaks at 2θ ≈ 25.99° and 47.86° are observed, which correspond to (002) and (100) diffraction peaks of rGO. This is because GO was reduced to rGO in situ after the treatment of 1,3-propanesultone. The stack size of SG was found to be 0.81 ± 0.03 nm, suggesting that the sulfonation of rGO is prone to occur on the edge of GO and in between the layers of GO, which facilitates the separation of rGO layers. For more clarification, the distance of the stacked layers of rGO was measured to be 0.53 ± 0.03 nm.

**Figure 2:**
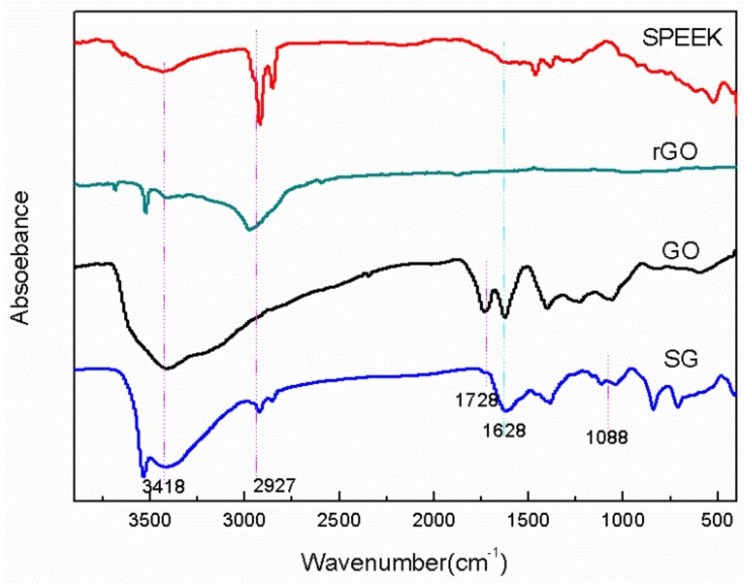
Transmission FTIR spectra of SPEEK, GO, rGO, and SG.

The FTIR spectra of rGO, GO, SG, and SPEEK is depicted in Figure 2. The transmission FTIR spectrum of the starting GO was predominantly composed of three peaks at 1728, 1628, and 1070 cm^−1^, which were attributed to the stretching vibrations of C=O, C=C (unoxidized SP^2^ CC bonds) and C–O, respectively. Compared with the GO spectrum, the absorption intensity of –OH, C–O, and C=O was weaker in the rGO spectrum. It was indicated that the content of these oxygen-containing functional groups significantly decreased. After GO sulfonation, the absorption peaks corresponding to O=S=O appeared at 1088 cm^−1^, which indicated the successful completion of sulfonation process. The spectra of both SPEEK and SG had strong absorption peak at 2927 cm^−1^, which was assigned to the saturated C–H. The four absorption peaks ranging from 1450 and 1600 cm^−1^ belonged to aromatic groups [32]. The absorption peak at 3418 cm^−1^ reflects the vibration of the O–H, and the peak of SG at 1628 cm^−1^ is stronger than that of SPEEK. This is consistent with the structure of the SG and SPEEK.

**Figure 3:**
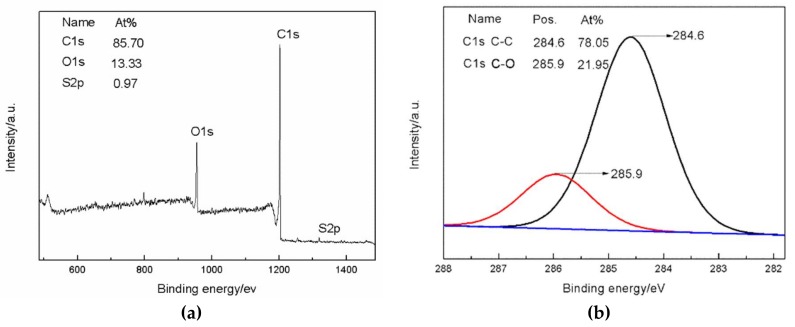
XPS Spectra of SG. (**a**) XPS wide-scan spectra of SG, (**b**) high-resolution C_1s_ XPS spectra of SG.

The chemical composition of GO and SG was confirmed by XPS, as shown in Figure 3. For SG, the XPS survey spectrum appears next to the band at 285 eV (C1s) and 532 eV (O1s) and a contribution at 200 eV due to S2p. The contents were as follows: C1s 85.70%, O1s 13.33%, and S2p 0.97% (Table 2). The formation of SG is also evidenced by the C1s high resolution spectrum (Figure 3b). The spectrum of SG can be deconvoluted into bands at 284.6 and 285.9, which are, respectively, assigned to C–C (sp^2^) and C–O functions. In the form of C elements, C–C is the largest in content, accounting for 78.05%, which is more than C–O and is consistent with the SG structure. Additionally, this is also consistent with the results measured by FTIR. All these results indicate that the sulfonated GO has sulfonic groups.

**Figure 4:**
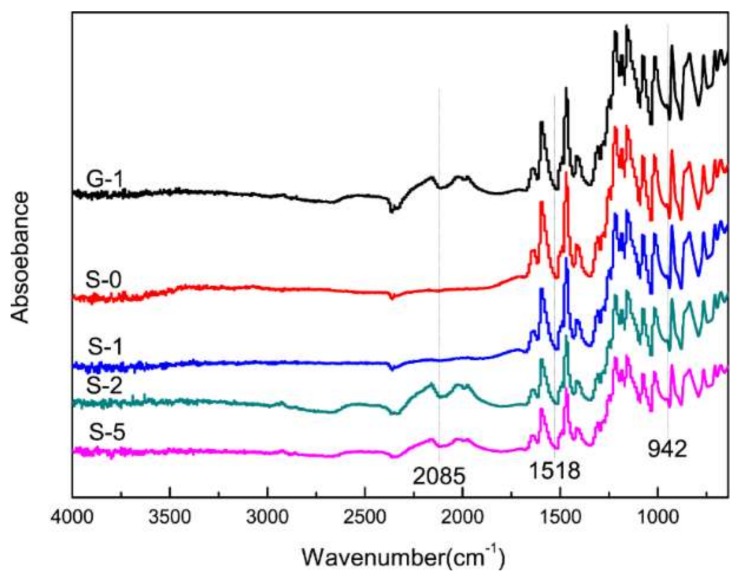
ATR-FTIR spectra of different composite membranes.

The ATR-FTIR spectra of the five PEMs are depicted in Figure 4. In the case of G-1, the bond at approximately 2085 cm^−1^ belongs to the vibrations of C=O or conjugated groups. For S-2 and S-5, the bond at 2085 cm^−1^ increases in intensity and is completely absent for S-0 and S-1. It is indicated that, with the increase of the amount of SG doping, oxygen-containing functional groups are obtained by the composite proton membrane. The vibrational bands assigned to the SO_3_-group are located at 942 cm^−1^, and the intensity of the peak decreases with the increase of SG doping. It is shown that hydroxyl groups and carboxyl groups in SG are combined with sulfonic acid groups in SPEEK to generate hydrogen bonding between them. Therefore, the consumption of sulfonate groups in the matrix (Less than 1%) increases with the rise of SG doping content.

### 3.2. IEC, Water Uptake, and Dissolvability

When the corresponding IEC value reached 2.1756, the DS value of S-0 sample was 75.86%, which was higher than the that in depicted in literature [10] (75%) and [18] (67%). It was indicated that the PEMs had high proton conductivity.

**Figure 5:**
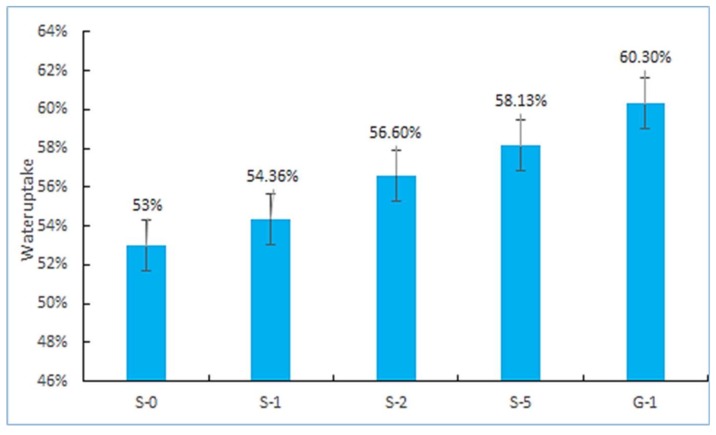
Water uptake of the different composite membranes.

The calculated water uptake was summarized in Figure 5. It was found that the water uptake of G-1 was higher than that of S-0. Generally speaking, the water uptake of membrane is related to its degree of electron accumulation and microstructure [33]. Since the electron accumulation of GO is higher than that of SPEEK, and the structure of GO with rich hydrophilic groups is more loose than SG, G-1 has higher water uptake [9]. The water uptake of SG/SPEEK PEMs increased obviously with the increase of the amount of SG doping due to the hydrophilic behavior of sulfonic groups. It is generally believed that the increased amount of water uptake can promote proton conduction at a relatively low scale (80%), and high water uptake above this value will cause excessive swelling rate and size change, and lead to the fuel cell invalidation [14,34,35]. Therefore, it is necessary to control the doping ratio of SG to obtain the best equilibrium state of water content and mechanical performance stability. The experimental results recorded in Table 3. S-1 and S-2 showed good dissolvability in methanol solution.

### 3.3. Proton Conductivity and Methanol Permeability

**Figure 6:**
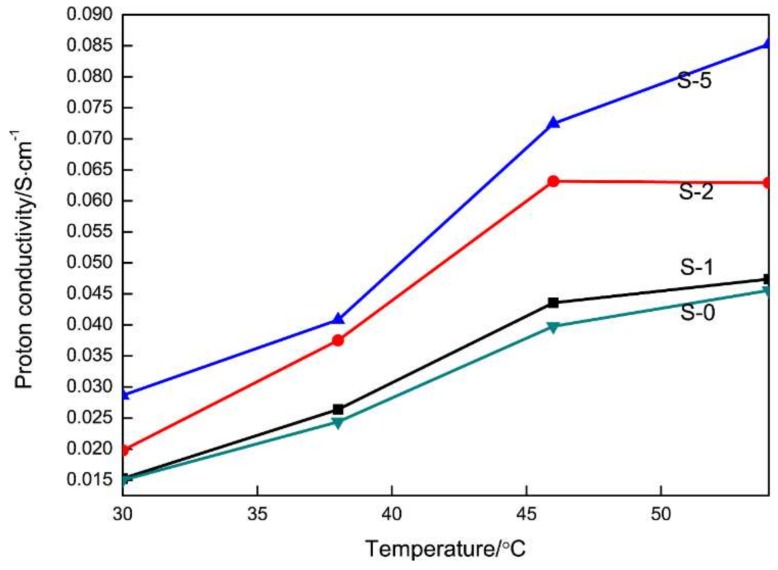
Proton conductivities of the different membranes.

As a key factor of PEMs, proton conductivity can determine the energy output and the working voltage of the fuel cell. The proton conductivity-temperature function of the composite PEMs is shown in Figure 6. It can be found that the increase of the amount of SG doping promotes proton conduction. Moreover, temperature is also the factor that influences the increase of proton conductivity.

Proton transport in PEMs is known to occur via two mechanisms. One is hopping mechanism, also known as the Grotthuss mechanism [36,37], in which the proton is passed down a chain of water molecules and transferred from one molecule to another through the formation and breaking of hydrogen bonds (proton hopping). The other is vehicle mechanism, in which the proton combines with the solvent molecules, producing H_3_O^+^ (hydronium ions) or CH_3_OH^2+^ complexes, which are then transported through the membrane. However, proton hopping and dispersion via transport mechanism and aqueous channel mechanism accelerates with the increase of the temperature. At the same temperature, the proton conductivity of the PEMs with more SG doping content becomes higher. This is because the well-dispersed SG enable the SPEEK substrates to possess a high surface area and a marked majority of oxygen-containing groups. Meanwhile, the SG nanosheets doping improves the microstructure of SPEEK and provides more exchange channels for protons.

Methanol permeability is an important parameter to determine the quality of a PEM for applications. Methanol molecules can permeate through relatively wide hydrophilic channels via vehicle mechanism in the process of proton conduction. The penetration of methanol in the membrane directly affects the efficiency of PEMFC, but the crossed-over methanol can cause catalyst poisoning, which consequently results in the reduction of the electrocatalytic activities of the cathode catalysts [38]. Therefore, lower methanol permeability is ideal for PEM [39].

**Figure 7:**
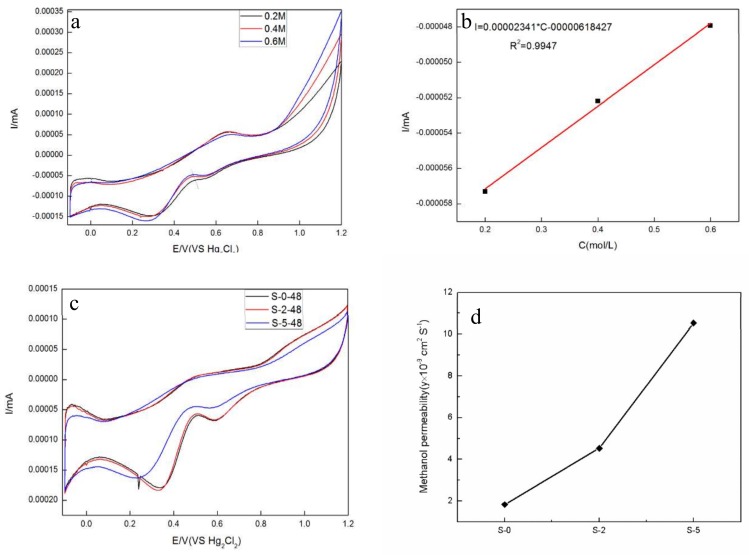
The electrochemical characterization of the SG/SPEEK PEMs. (**a**) CV curves at different methanol concentrations; (**b**) the peak currents versus concentrations of methanol; (**c**) CV curve for cell B at RT; (**d**) the value of PEMs’ methanol permeability.

It can be obtained from Figure 7a,b that the peaks of the cyclic voltammetry curves have a good linear relationship with the concentration of methanol (correlation coefficient 0.9947). The curves for methanol diffusion are displayed in Figure 7c. Additionally, the methanol permeability increases with the increase of the SG doping amount. The reason is that the internal sulfonic acid groups are in the form of hydrophilic ion clusters; protons in the form of H_3_O^+^ enable the connection between the channels to complete the proton transfer. In this environment, the methanol molecules can also pass through the channel. The methanol permeability of PEMs after the 48 h permeation was calculated by Formula (5) as shown in Figure 7d. It indicated that the PEMs, whose methanol permeability is better than that of the previously reported SPEEK (1.25 × 10^−^^8^ cm^2^ S^−1^) [28], is suitable for PEMFC. Compared with the other PEMs, S-2 displayed good dissolvability, preferable proton conductivity, and superior alcohol resistance.

### 3.4. Thermal Stability and Morphology Study

**Figure 8:**
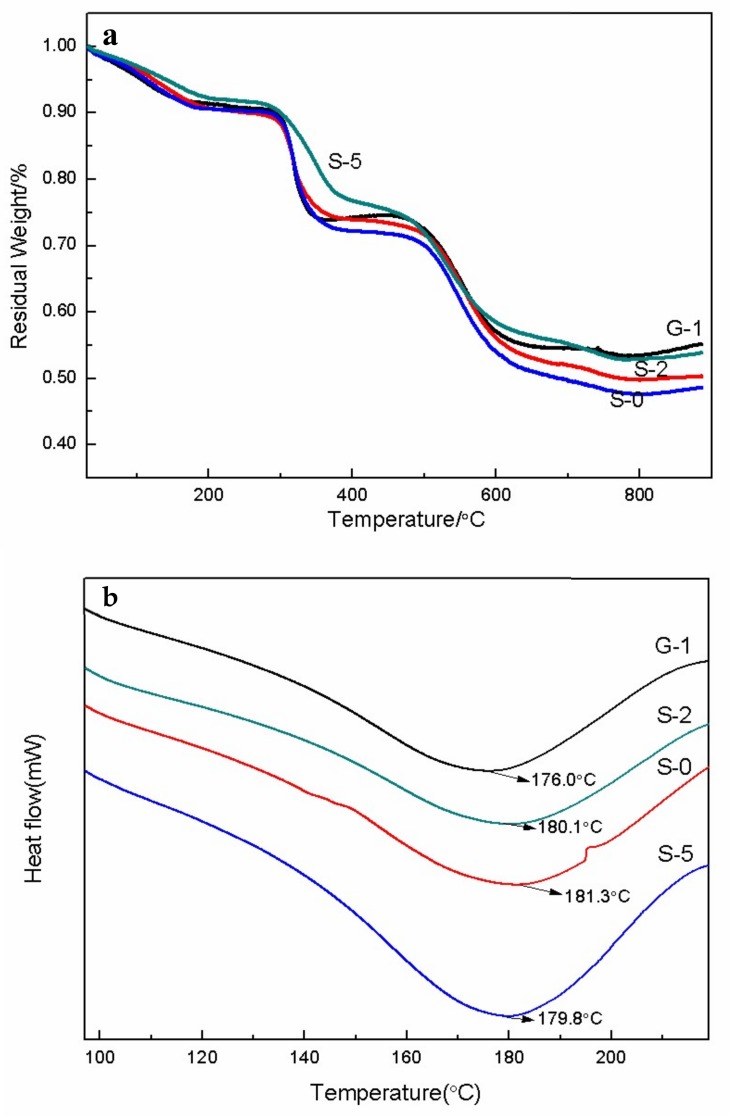
The thermal analysis of the (**a**) TGA curve and (**b**) DSC curve of four types of PEMs.

To investigate the thermal stability of PEMs, TGA analysis was carried out (Figure 8a). The TGA thermograph of PEMs samples showed that a mass loss of 10% was within the heating range of 30–100 °C due to the evaporation of water adsorbed on the membrane surface. From 285 to 380 °C, a sharp weight loss was recorded due to the degradation of sulfonic acid groups of the samples. From 380 to 540 °C, all the curves declined slowly. For example, there was only 2.3% weight loss of S-0 sample in this heating range. Moreover, the decomposition of polyimide chains resulted in another weight loss. In contrast to G-1, S-1, and S-0, the introduction of SG and GO can effectively improve the thermal stability of the membranes, and the effect of SG is more obvious. Generally speaking, all membranes maintain a relatively stable weight at normal PEMFC operating temperatures. It is indicated that these SG/SPEEK membranes are more suitable for PEMFC. Figure 8b showed the DSC thermograms (2nd heating) for PEMs. The glass transition temperature (Tg) of S-0 occurs approximately at 181.3 °C. The incorporation of GO fillers decreased the Tg values of SPEEK by significantly reducing the crystallinity of SPEEK backbones. Therefore, the Tg value of G-1 was 176.0 °C. There is nearly no shift in the Tg between SPEEK and SG/SPEEK membrane. It can be seen that the thermal stability of SG/SPEEK membranes is maintained.

**Figure 9:**
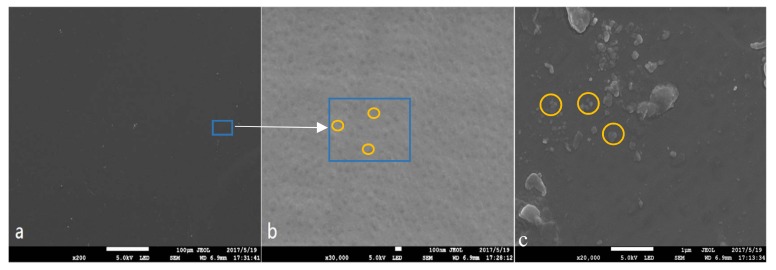
Morphologies of the surface of the membranes (**a**,**b**) S-0; (**c**) S-2. Arrows show the particles of the surface of membranes.

**Figure 10:**
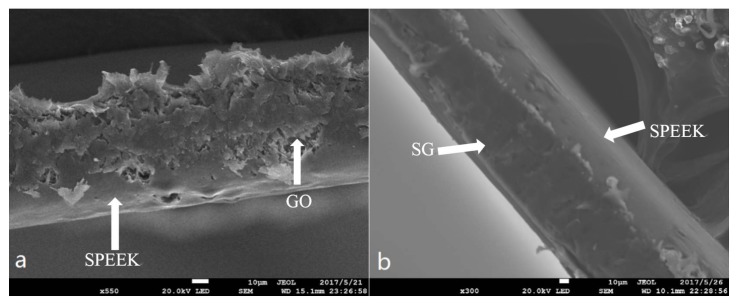
Morphology of the cross sections of PEMs (**a**) G-1; (**b**) S-2. Arrows show the fillers and the matrix of membranes.

**Figure 11:**
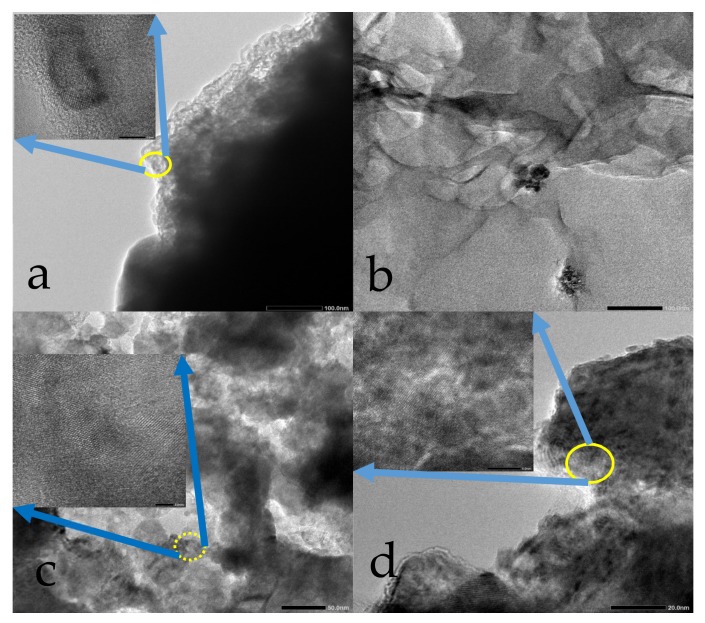
Transmission electron microscopy images of GO (**a**), SG (**b**), G-1 (**c**), and S-1 (**d**).

The SEM morphologies of the PEMs surface are shown in Figure 9. It is found that the SPEEK membrane has a smooth surface morphology without any obvious pore (Figure 9a). Under a high magnification, there were dispersed nanoparticles with the size of ~100 nm uniformly depositing on the surface (Figure 9b). After SG doping into SPEEK membrane, the basic roughness of the S-2 was improved, and some lager particles appeared on it (Figure 9c). The sulfonic acid groups on the surface of SG are linked to GO through a benzene ring; SG are scattered in the SPEEK base membrane to increase the gap between the segments. Figure 10 shows the SEM micrographs of the cross sections of PEMs. It was observed that the GO lamellae filler in the composite PEMs was well interconnected with the SPEEK substrates in G-1 sample (Figure 10a). Meanwhile, there was a more compact cross-section structure in S-2 sample. It is indicated that SG has better interface wettability, chemical compatibility, and crosslink behavior in the SPEEK substrate [40]. The morphologies of GO and SG were observed by TEM as shown in Figure 11a,b. GO had typically 2D sheet-like structure [41,42]. The GO sheets were obviously overlapped. On the contrary, the SG nanosheets were well dispersed as shown in Figure 11b. The SGO nanosheets show wavy and well exfoliated layered structure resulting from hydrophibic sulfonic acid group modification on the GO surface. Compared to G-1, the SG was dispersed homogeneously on the SPEEK matrix in S-1, as shown in Figure 11c,d.

## 4. Conclusions

We utilized a sulfonation method to prepare a series of SG/SPEEK composite membranes. An electrochemical study revealed that the PEM exhibited excellent proton conduction with the increase of the SG doping amount. The SG/SPEEK composite membrane (loaded with 2 wt % of SG) had proton conductivities of 0.063 S cm^−1^ at 54 °C, which is 1.54 times higher than that of the SPEEK (0.041 S cm^−1^) membranes. The methanol permeabilities of SG/SPEEK (loaded with 2 wt % of SG) and SPEEK membranes were 1.834 × 10^−9^ and 4.537 × 10^−9^ cm^2^ S^−1^, respectively. Compared with the control groups of the GO-doped SPEEK and SPEEK membranes, the SG/SPEEK composite membranes have better PEMs behaviors in the field of water uptake and thermal stability. Therefore, as a novel PEMs–SG/SPEEK composite membrane has promising commercial application prospect.

## Figures and Tables

**Table 1:** The list of different membranes.

Sample	GO/g	SG/g	SPEEK/g
G-1	0.012	0	1.188
S-0	0	0	1.200
S-1	0	0.012	1.188
S-2	0	0.024	1.176
S-5	0	0.060	1.140

**Table 2:** Atomic percentages of different elements.

Samples	C1s (at %)	O1s (at %)	S2p (at %)	C/O
GO	67.49	32.51	0	2.08
SG	85.70	13.33	0.97	6.43

**Table 3:** Statistical results of the PEMs’ dissolvability in different solvents.

	G-1	S-0	S-1	S-2	S-5
chloroform	+	+	+	+	+
DMF	+	-	-	-	+
methanol	-	-	/	/	-
NMP	+	+	+	+	+
acetone	/	/	/	/	-
DMSO	+	-	-	+	+

+: Dissolve; -: slightly soluble; /: undissolved.
